# Experimental Study on Tensile Performance of FRP Tendons/Cables with Varied Bond Anchorage Factors

**DOI:** 10.3390/ma17010004

**Published:** 2023-12-19

**Authors:** Xing Zhao, Lanjinhua Meng, Sihao Li

**Affiliations:** Department of Civil Engineering and Airport Engineering, Nanjing University of Aeronautics and Astronautics, Nanjing 211106, China; jinhua_meng@163.com (L.M.); li511246260@163.com (S.L.)

**Keywords:** FRP tendons, bonded anchorages, thickness, bonding length, strain distribution

## Abstract

FRP tendons and cables are increasingly being used in civil engineering structures due to their high strength-to-weight ratio and corrosion resistance. The bond anchorage factors, which characterize the bond strength between the FRP tendon/cable and the surrounding materials, play a critical role in determining the overall performance of the system. In this study, a series of tensile tests were conducted on FRP tendons/cables with different bond anchorage factors to evaluate their load-carrying capacity, load–displacement curve, and strain distribution. The study considered different types and surface shapes of FRP tendons/cables, and determined the influence of anchoring length, bonding medium type, and bonding medium thickness on the performance. The strain distribution of FRP tendons/cables at the anchorage end gradually increased along the loading section to the free end. A stress analysis model of the anchoring section was proposed and found to be consistent with the test results.

## 1. Introduction

Fiber-reinforced plastic/polymer (FRP) has excellent properties of lightweight, high-strength, and corrosion resistance [1,2,3,4,5,6]. The use of FRP tendons/cables instead of steel cables in bridge engineering has broad application prospects [7,8]. To connect to other structure members, anchorages are used for FRP tendons/cables. A large number of tests on anchorage systems of FRP tendons have been conducted [1,3,6,9,10,11,12,13,14,15,16,17,18]. The commonly used anchoring methods for FRP tendons mainly include bonded anchoring, mechanical anchoring, and composite anchoring [6]. Bonded anchoring devices transfer shear stress through the bonding force, friction force, and chemical bonding force between the bonding medium and FRP tendons, which can avoid transverse shear failure of FRP tendons and have a simple structure and high anchoring efficiency.

There are two commonly used bonding media: resin and cement-based materials [19]. The bonding strength between resin and FRP tendons is very high, the pouring process is simple and the curing cycle is fast. Huang et al. [20] showed that the relative slip of the specimen was smaller and the shear stiffness was greater when using resin with a higher elastic modulus to anchor basalt fiber-reinforced plastic/polymer (BFRP) tendons. Some scholars and engineers have modified the resin to improve its anchoring performance. In the anchorage used in the Stork Bridge in Switzerland, engineers mixed Al_2_O_3_ particles into the resin filling material in the anchorage, allowing the elastic modulus of the bonding medium to change in a gradient, thereby avoiding stress concentration at the open end of the anchorage [8]. Although resin has a low curing time, its thermal stability and creep performance are poor. Zhuge et al. [21] proposed that when resin is used as the load transfer medium for anchors, the creep of the resin will significantly reduce the load-bearing capacity of the anchor. The corrosion resistance of the resin is also poor, which can lead to performance degradation of FRP anchoring devices in corrosive environments. Therefore, under corrosive environments and repeated loading conditions, resin is not an ideal bonding medium. Some scholars have proposed using cement-based materials with good corrosion resistance as the bonding medium [22]. 

The size parameters of the anchor also have a significant impact on the anchoring performance. For bonded anchors, a sufficiently long anchorage length is necessary to ensure that the FRP tendons do not slip, leading to early failure of the anchor. Ali et al. [23] used a anchoring length of 68 *d*_f_ when anchoring CFRP strands with a diameter of 7.5 mm using epoxy resin. Fang et al. [24] found that a bonding length of 25 *d*_f_ (*d*_f_ is the diameter of the FRP tendon) can ensure the full strength of the carbon fiber reinforced plastic/polymer (CFRP) tendon for 10 mm diameter CFRP tendons anchored with high-performance concrete (reactive power concrete, RPC), but a bonding length of 35 *d*_f_ cannot guarantee the CFRP bar to be pulled apart when the bonding medium is ordinary concrete and resin mortar. Fang et al. [25] used a 40 *d*_f_ anchoring length when anchoring CFRP strands with a diameter of 4.17 mm using RPC. Saeed et al. [15] used micro expansion concrete to anchor CFRP tendons with diameters of 10 mm and 13 mm and recommended the minimum anchoring lengths of 32 *d*_f_ and 30 *d*_f_, respectively. The thickness of the bonding medium also affects the anchoring performance. Huang et al. [20] proposed that the greater the thickness of the bonding medium of epoxy resin, the greater the ultimate tensile strength of the anchor specimen, because only a sufficiently thick bonding medium can ensure sufficient shear stiffness. This conclusion is similar to that of Puigvert et al. [14]. According to the fatigue and creep tests, anchors with thicker adhesive medium of low viscosity polyamine cured epoxy exhibit better fatigue and creep performance [26]. Saeed et al. [15] proposed the concept of Rod to anchor area ratio, pointing out that when the ratio is larger (the medium thickness is smaller), it can limit the deformation of the grouting inside the sleeve, increase the slip stiffness, and thus improve the tensile strength of the anchor. The bond anchorage factors, which characterize the bond strength between the FRP tendon/cable and the surrounding materials, play a critical role in determining the overall performance of the system.

In this paper, the tensile and anchoring performances of bond anchorage of BFRP and CFRP tendons with smooth and deeply ridded surfaces were investigated using different types of resins and ultra-high-performance concrete as bonding media. The effect of bond anchorage factors like anchoring lengths and bonding media thicknesses were also evaluated. The anchoring performances and mechanism of different bond anchorage factors were studied to obtain appropriate anchoring parameters for different FRP tendons.

## 2. Experimental Program

### 2.1. Materials

The FRP used in this paper included basalt fiber-reinforced polymer (BFRP) and carbon fiber-reinforced polymer (CFRP) tendons/cables in the form of BFRP bars, both of which were produced by Jiangsu GMV Co. (Nanjing, China) with a nominal diameter of 6 mm. The BFRP and CFRP tendons were made of basalt fiber GMV-BF1200 (1200 tex of ≥2500 MPa) and carbon fiber M40JB-12k (≥4400 MPa) from Jiangsu GMV Co. The fiber contents of BFRP tendons and CFRP tendons were measured to be 65% and 68% by volume using the density measurement method, respectively. The matrix used in FRP tendons was epoxy resin. The equivalent diameters of the smooth and deep-ribbed FRP bars/tendons were 6.0 mm and 5.8 mm, respectively. The bonded anchorage system consisted of FRP tendon, steel sleeve and bonding medium. Three types of bonding medium were studied. They were Ultra-High-Performance-Concrete (UHPC) cement paste produced by Hunan Guli Engineering New Materials Co., Ltd. (Changsha, China), L-500 epoxy resin produced by Shanghai Sanyo Resin Co., Ltd. (Shanghai, China) and JZ epoxy resin produced by from Nanjing Mankate Science & Technology Co., Ltd. (Nanjing, China). The steel sleeves were seamless steel pipes with an outer diameter of 20 mm, a wall thickness of 3 mm and a tensile strength of 475 MPa provided by the manufacturers. The UHPC cement paste was mixed according to a water–cement ratio of 0.2, and the measured 28 days compressive strength of the cement paste was 15.4 MPa. The measured tensile strengths of the L-500 resin and JZ resin were 46.07 MPa and 30.18 MPa, respectively. The tensile strengths were similar to epoxy matrix resins in the literature [27].

### 2.2. Specimens Preparation

**Figure 1:**
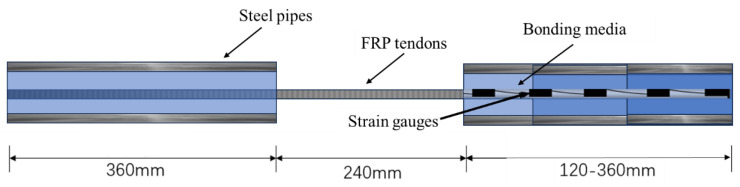
Geometry configuration of specimens.

At both ends of the FRP tendons, seamless steel pipes were used as sleeves, and L-500 resin and UHPC cement paste were used as bonding medium between the FRP tendons and the steel pipes. Figure 1 shows the dimensions of the specimens, with one end anchor length of 360 mm to prevent anchor failure and one end anchor length of 120 mm to 360 mm to study the strain distribution in the anchorage.

The steel pipes were polished with coarse sandpaper and a brush. The surface of the FRP tendons in the anchor section was polished every 30 mm and wiped with ethanol to place five strain gauges at the polished position. The FRP bars/tendons were sand-coated to enhance the friction between the FRP and the bonding medium. The specimens using resin as the bonding medium were cured for 7 days, and the specimens using UHPC as the bonding medium were cured for 28 days.

The parameters of the test specimens are listed in Table 1. The surface shapes of FRP bars/tendons used were divided into two types: deep-ribbed and smooth surface. According to ASTM D7205/D7205M, the fixed section length of the anchor is set to 360 mm. The length of the anchoring section is determined to be 20 *d*_f_, 25 *d*_f_, 30 *d*_f_, 35 *d*_f_, and 40 *d*_f_ (*d*_f_ refers to the diameter of FRP tendons) referring to the literature [15,23,24,25]. The experiment used three bonding media: JZ resin, L-500 resin, and UHPC mortar. Three filling thicknesses of 2 mm, 3 mm, and 4 mm were used. The centricity of the tested bars in the steel pipes was ensured by wrapping adhesive tape to fix the alignment.

### 2.3. Test Setup

The static load tensile tests were conducted using a WE-1000 universal testing machine and the static strain acquisition instrument DH3818-2 produced by Dong Hua Testing Technology Co., Ltd. (Jingjiang, China). The load cell used in the mechanical testing equipment was a hydraulic universal testing machine with a maximum load cell of 1000 kN and measurement range of 2~100%. The loading rate was 2 mm/min. During the loading process, the strain was collected every 2 kN of the load, and the states of the specimen during the test were recorded.

## 3. Results and Discussion

### 3.1. Load-Carrying Capacity

**Figure 2:**
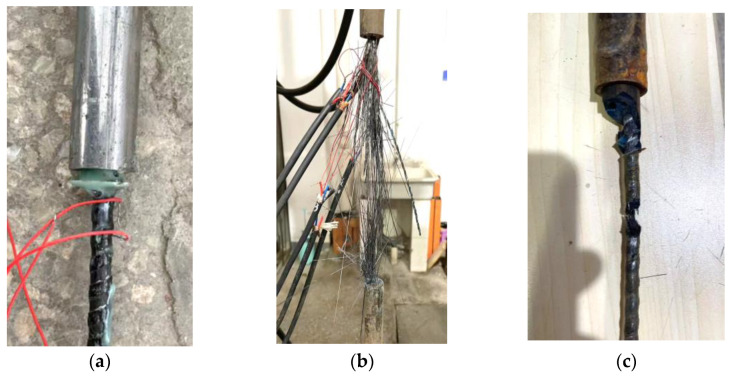
Failure patterns of the specimens. (**a**) Pullout failure. (**b**) Rupture failure. (**c**) Shear failure.

The typical failure patterns of the specimens in the tensile tests were pullout failure, rupture failure and shear failure, as shown in Figure 2. The failure modes of specimens are listed in Table 2. As the strength and ultimate load of CFRP were higher than BFRP, the CFRP specimens were more likely to have pullout failure. Shear failure was observed for some CFRP specimens as a result of anchor eccentricity and brittleness of CFRP. 

The results of the static tensile test are shown in Table 2. Only one sample was tested per condition in some cases as the effect of the factors can be compared with more than one condition. The ultimate strength of some specimens was not recorded due to premature slip failure. The surface shape of FRP tendons has a significant impact on the anchoring performance. The specimens of BFRP tendons with smooth surfaces anchored with L-500 resin in Table 2 underwent slip failure. When the anchoring length is 20 *d*_f_, the ribbed specimens with JZ resin all exhibit slip failure. When the anchoring lengths are 25 *d*_f_ and 30 *d*_f_ with JZ resin as specimens BRL-25 *d*_f_-3 and BRL-30 *d*_f_-3, one of the three ribbed specimen experienced slip. The ribbed specimens with anchoring lengths of 35 *d*_f_ and 40 *d*_f_ of JZ resin (BRL-35 *d*_f_-3 and BRL-40 *d*_f_-3) exhibited tensile rupture failure and significantly higher tensile strength than other specimens. With L-500 resin and UHPC as the bonding mediums, the failure mode of deep-ribbed BFRP specimens of all anchoring lengths was tensile rupture failure. Their tensile strength increased with the increase of anchoring length. The increasing the anchorage length can provide a larger load transfer area and ensure sufficient bonding force. However, the economy and operability of the anchorage also need to be considered. The minimum anchorage length required to achieve the maximum tensile strength of FRP tendons without premature anchorage failure. That is to ensure that the FRP specimen can undergo tensile failure in the free section, while avoiding premature sliding or shear failure in the anchoring section. The specimens with an anchorage length less than 30 *d*_f_ experienced slip, while the specimens with an anchorage length greater than 30 *d*_f_ exhibited tensile failure. It indicates that the stability of anchorage performance can be guaranteed when the anchorage length is 30 *d*_f_ for the deep-ribbed BFRP tendons. 

There are significant differences in the mechanical properties of different bonding media, and there are also differences in the bonding force between them and FRP tendons. The average tensile strength of the specimens using L-500 resin is higher than that of the specimens using JZ resin under the three anchoring lengths. This is because the elastic modulus of L-500 resin is larger, which increases its ability to resist deformation when transmitting tension and stronger bonding with BFRP tendons. When the thickness of the bonding medium was 4 mm, the average tensile strength of the specimens was not significantly different when L-500 resin and UHPC slurry were used to anchor BFRP tendons. It can be considered that the anchoring performance of the two bonding media is not significantly different at this time.

Under the three anchoring lengths, the average tensile strength of the specimens with a bonding media thickness of 3 mm is slightly higher than that with a thickness of 4 mm, and the difference is about 10%. The reason may be that when the resin layer is thicker, its deformation may also increase, leading to a decrease in anchoring performance [17,20,21]. When the bonding media thickness is 3 mm and 4 mm, the anchoring performance is also basically the same, but a minimum thickness of 3 mm needs to be ensured.

### 3.2. Load–Displacement Curve

**Figure 3:**
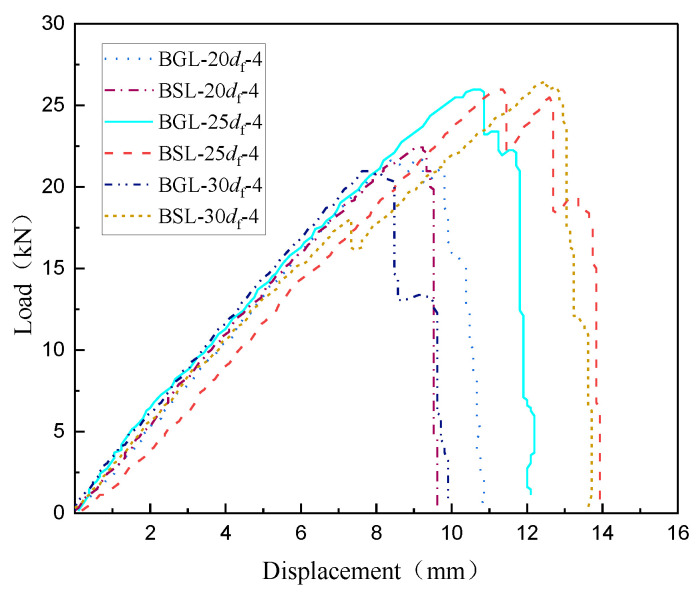
Load–displacement curves of BFRP tendons using different bonding media.

Figure 3 shows the load displacement curve of BFRP tendons using different bonding media. It can be seen that when the anchoring length is the same, the displacement value of the specimen anchored with UHPC is smaller than that of the specimen anchored with L-500 resin. Moreover, in the decreasing section of the load, the load of the specimens BSL-20*d*_f_ and BSL-30*d*_f_ using L-500 resin shows a linear decrease, while the load curve of the specimen BGL-20*d*_f_ and BGL-30*d*_f_ using UHPC slurry shows a fluctuating decrease in the decreasing section. This is because compared to resin, UHPC has a higher elastic modulus and stronger resistance to deformation, so its load will not decrease sharply. An exception appeared in BSL-25*d*_f_ because the specimen showed multiple fractures during the loading.

**Figure 4:**
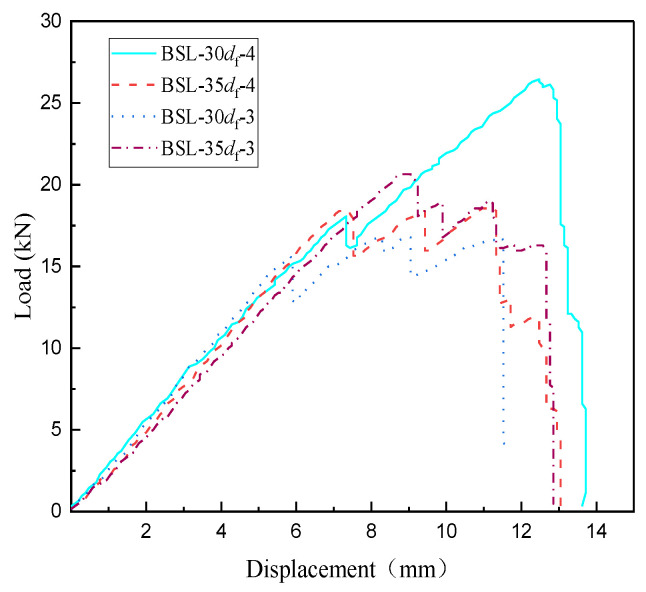
Load–displacement curve of BFRP tendons using different thickness of bonding medium.

Figure 4 shows the load–displacement curve of BFRP tendons using different thickness of bonding medium. It can also be seen that BSL-35*d*_f_-4 with L-500 resin of a thickness of 4 mm exhibits larger load of specimen BSL-35*d*_f_-3 with a thickness of 3 mm. This may be because the thicker bonding medium provides a larger force transfer area and also reduces the radial stress in the anchoring area.

### 3.3. Strain Distribution of Anchoring Section

**Figure 5:**
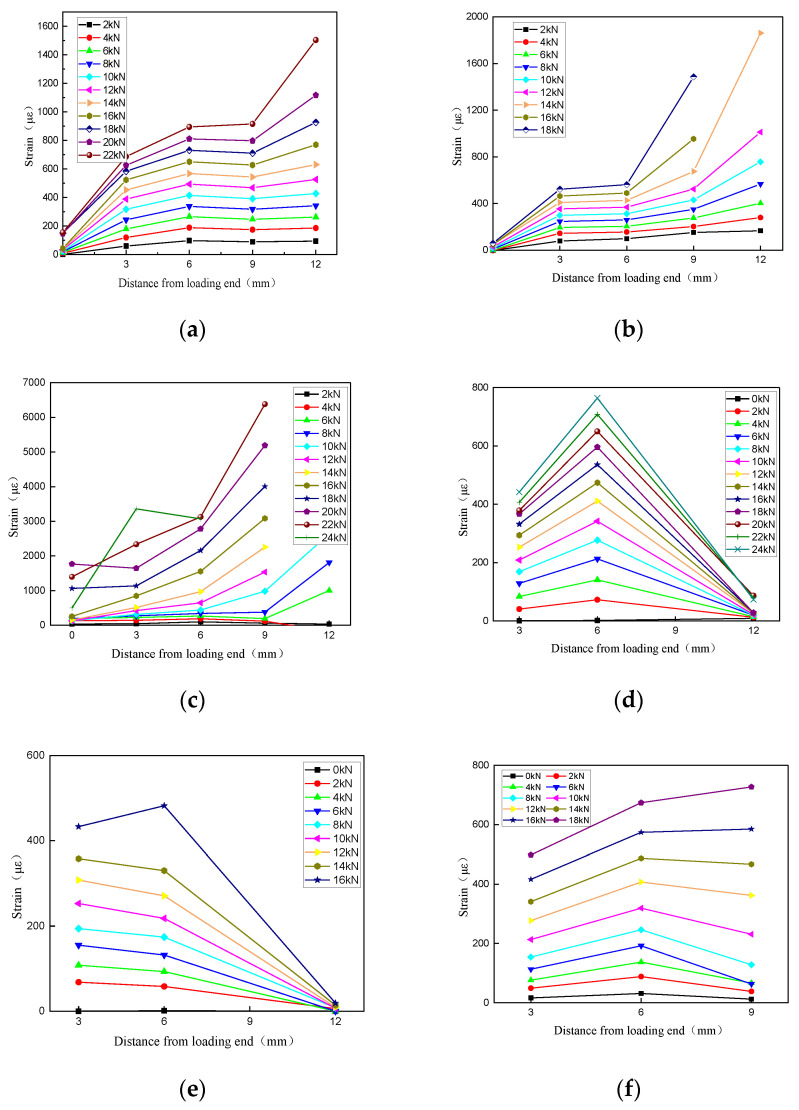

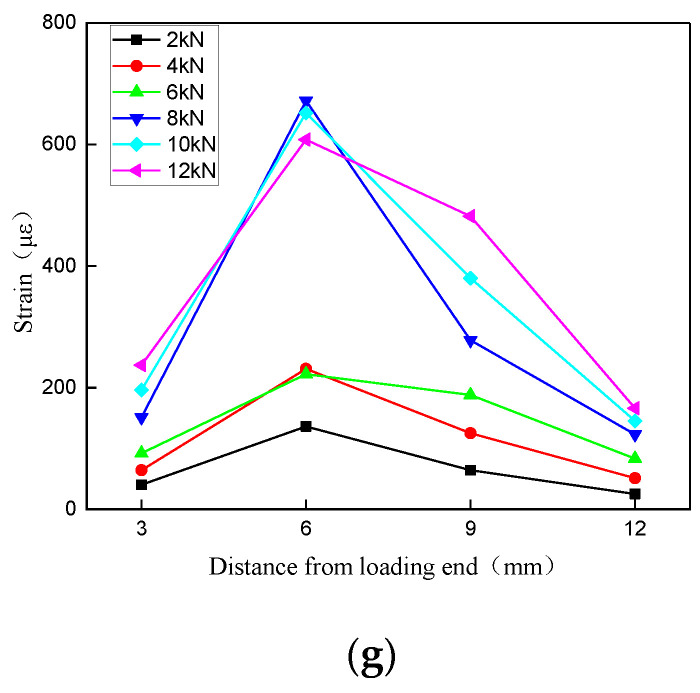
Strain distribution of some FRP tendons in the anchorage section. (**a**) BRL-40*d*_f_-3. (**b**)BRL-35*d*_f_-3. (**c**) BGL-30*d*_f_-4. (**d**) BSL-40*d*_f_-3. (**e**) BSL-30*d*_f_-3. (**f**) BSL-30*d*_f_-4. (**g**) BSY-30*d*_f_-4.

Figure 5 shows the strain distribution of some FRP tendons in the anchorage section. During the loading process, the strain of FRP tendons gradually increases from the loading end to the free end. This is because the specimens are clamped at the loading end, and the slip between the FRP tendons and the bonding medium first occurs in the middle far from the loading end. As the slip increases, the strain gradually increases. When the test force approaches the ultimate load, the strain gauge far from the loading end will fail due to excessive slip, and the strain collection instrument will not be able to collect data.

Comparing Figure 5a,b, it can be observed that the strain distribution of specimen BRL-40*d*_f_-3 is smoother, indicating that a longer anchorage length provides a larger stress transfer area and a smaller average strain. The strain distribution of specimen BSL-30*d*_f_-4 with a bonding medium thickness of 4 mm (Figure 5f) is also smoother than that of specimen BSL-30*d*_f_-3 with a thickness of 3 mm (Figure 5e). In addition, the strain of specimens using UHPC is significantly greater than that of specimens using resin, because the slip interface is different. The failure mode of specimens using UHPC is tensile failure or the interface between FRP tendons and UHPC slides. The strain gauge is attached to the surface of FRP tendons, so the strain is significantly greater than that of specimens with adhesive medium slip failure. At the same time, the elastic modulus of UHPC is higher, and the mechanical interlocking and friction force between UHPC and FRP tendons is also greater, which also affects the distribution of strain. Similarly, the strain decrease trend in specimen BSY-30*d*_f_-4 (Figure 5g) with a smooth surface near the free end is significantly greater than that of specimen BSL-30*d*_f_-4 (Figure 5e) with a deep-ribbed surface thread. This is also due to the difference in friction between the two and the bonding medium, resulting in different interfacial slip values. The strain distribution in Figure 5d–g decreases from the loading end to the free end, possibly due to the eccentricity effect that occurs when anchoring FRP tendons, or the uneven mixing of the bonding medium, as well as the differences in strain gauge adhesion. It can be seen that the anchorage length, surface shape of FRP tendons, type and thickness of bonding medium have important effects on the strain distribution at the anchorage end of FRP tendons.

## 4. Stress Analysis and Prediction of Anchoring Section

**Figure 6:**
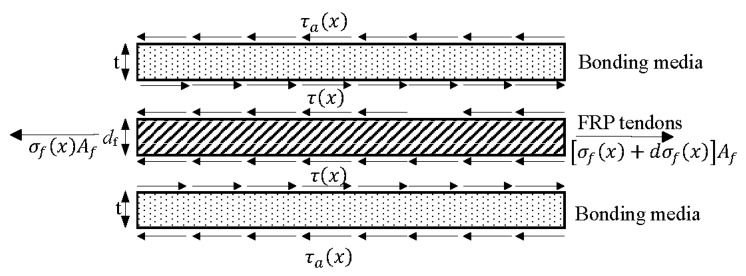
Schematic diagram of the stress at the interface between FRP tendons and bonding media.

Figure 6 is a schematic diagram of the stress at the interface between FRP tendons and bonding media. The stress analysis for the anchoring section is predicated on three assumptions: (1) linear elastic behavior of FRP tendons, steel sleeves, and bonding media; (2) negligibility of steel sleeve deformation due to its high stiffness; (3) solely shear deformation of the bonding medium while transmitting shear force; and (4) stress and displacement of free end of the FRP tendons and bonding media were zero while the stress of loading end of the FRP tendons were the same stress of FRP tendons in the gage length. The stress equilibrium conditions of FRP tendons are considered.

(1) $$\sigma_{f}(x)A_{f}+\pi d\tau (x)dx=\left[\sigma_{f}(x)+d\sigma_{f}(x)\right]A_{f}$$

(2) $$\tau (x)=\frac{d_{f}}{4}\frac{d\sigma_{f}(x)}{dx}$$

In the above equation, $\sigma_{f}(x)$ represents the axial stress at the longitudinal *x* position of the FRP tendons, τ(x) is the stress at the longitudinal *x* position of the FRP tendons bonding medium interface, in MPa, and $A_{f}$ is the cross-sectional area of the FRP tendons, in mm^2^.

According to the force balance relationship between the internal and external interfaces of the bonding medium:

(3) $$\pi d\tau (x)=\pi (d_{f}+2t)\tau_{a}(x)dx$$

(4) $$\tau (x)=\frac{d+2t}{d}\tau_{a}(x)$$

$\tau_{a}(x)$ is the stress at the longitudinal *x* position of the interface between the steel sleeve and the bonding medium, in MPa. $d_{f}$ is the diameter of the FRP bars/tendons, *t* is the thickness of the bonding medium, all in mm.

According to the deformation coordination relationship, the relative slip between the FRP tendons and the bonding medium is:

(5) $$u(x)=u_{f}(x)-u_{a}(x)$$

Taking a micro segment *dx*, consider:

(6) $$du(x)=\left[\varepsilon_{f}(x)-\varepsilon_{a}(x)\right]dx$$

u(x) is the relative slip of the interface along the longitudinal *x* position, $\varepsilon_{f}(x)$ is the longitudinal strain of the FRP tendons, and $\varepsilon_{a}(x)$ is the longitudinal strain of the bonding medium.

Taking the derivative on both sides of Equation (6) yields:

(7) $$\frac{du(x)}{dx}=\varepsilon_{f}(x)-\varepsilon_{a}(x)$$

Assuming that the shear stress is linearly distributed along the radial direction of the anchoring section, the average shear stress of the bonding medium can be expressed as:

(8) $$\bar{\gamma }=\frac{\tau (x)+\tau_{a}(x)}{2G}$$

Shear modulus G (MPa) of the bonding medium can be obtained by combining the above methods:

(9) $$\frac{u(x)}{t}=\bar{\gamma }=\frac{d+t}{d+2t}\frac{\tau (x)}{G}$$

For the shear stress at the interface of the anchoring section bonding medium—FRP tendons, it can be obtained:

(10) $$\tau (x)=\frac{d+2t}{d+t}\frac{G}{t}(C_{1}e^{x}+C_{2}e^{-x})$$

**Figure 7:**
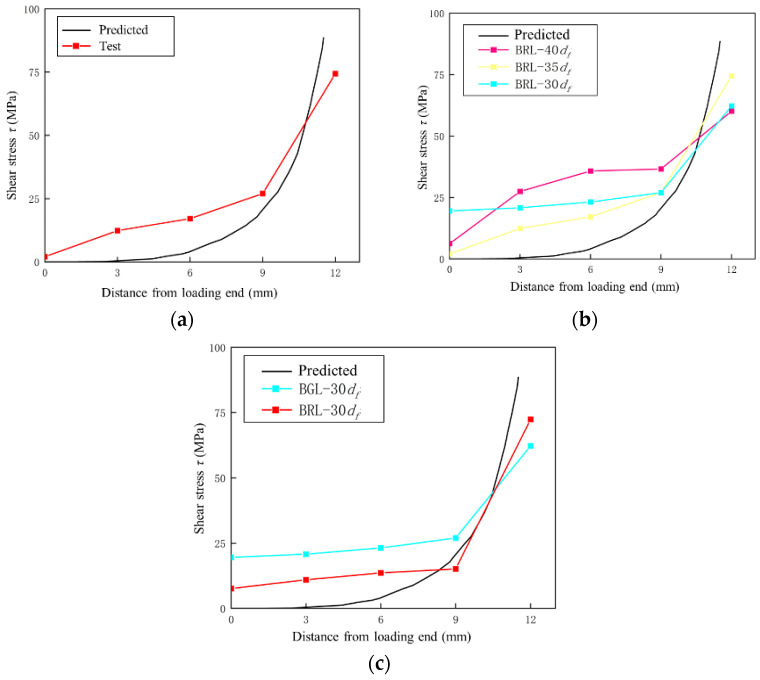
Shear stress distribution at the anchorage end. (**a**) BRL-35 *d*_f_-3. (**b**) Specimens with different anchoring lengths. (**c**) Specimens with different bonding media.

Figure 7 shows the comparison between the shear stress distribution at the anchorage end of some selected specimens and the theoretical values. The experimental shear stress data were calculated by the strain distribution from the strain gauges multiplying the modulus of the FRP tendons. The theoretical values are obtained from Equation (10), and it can be seen that the stress trend of the anchorage section of the specimens under different experimental parameters is relatively consistent, and the difference is not significant. Although there are certain differences in the numerical values between the two cases, the curve trend is relatively consistent. Considering the influence of defects on the strain distribution on the surface of FRP tendons and bonding media, it can be considered that there is a certain degree of consistency between the two.

## 5. Conclusions

In this paper, static tensile tests were conducted on BFRP and CFRP tendons using the bonded anchoring method. The bond anchorage factors, surface shapes of FRP tendons/cables, anchoring length, bonding medium type, and bonding medium thickness, were studied to find their influences on the overall system performance. Based on the results, the following conclusions can be obtained:

1. It is crucial to ensure a sufficiently long anchoring length to prevent premature sliding of the FRP specimens. For anchoring BFRP and CFRP tendons with JZ resin, L-500 resin, and UHPC slurry, a minimum basic anchoring length of 30 times the diameter of the tendons should be maintained.
2. The bonding strength of L-500 resin or UHPC was significantly stronger compared to that of JZ resin. UHPC exhibits better bonding performance when anchoring CFRP tendons. Additionally, all three bonding media must have a minimum filling thickness of 3 mm.
3. FRP tendons with smooth surfaces experienced weak mechanical biting force and friction force with the resin, resulting in sliding failure of the specimens. When using UHPC to anchor CFRP tendons, attention should be given to avoiding damage caused by excessive internal stress on the CFRP due to the high ultimate load.
4. The peak strain of FRP tendons at the anchorage end was observed near the middle of the specimen and close to the free end. The strain distribution gradually increased along the loading section towards the free end. The type and thickness of the bonding medium have a significant influence on the slip development of FRP tendons in the anchoring section, thus affecting the strain distribution.

## Figures and Tables

**Table 1:** Summary of the number of specimens taken into account by different variables.

Specimen ID	FRP Type	Bonding Length (mm)	Bonding Medium Type	Surface Type of Tendons	Thickness of Medium (mm)
BRL-20 *d*_f_-3	BFRP	120	JZ	Deep-ribbed	3
BRL-25 *d*_f_-3	BFRP	150	JZ	Deep-ribbed	3
BRL-30 *d*_f_-3	BFRP	180	JZ	Deep-ribbed	3
BRL-35 *d*_f_-3	BFRP	210	JZ	Deep-ribbed	3
BRL-40 *d*_f_-3	BFRP	240	JZ	Deep-ribbed	3
BSL-20 *d*_f_-4	BFRP	120	L-500	Deep-ribbed	4
BSL-25 *d*_f_-4	BFRP	150	L-500	Deep-ribbed	4
BSL-30 *d*_f_-4	BFRP	180	L-500	Deep-ribbed	4
BSL-35 *d*_f_-4	BFRP	210	L-500	Deep-ribbed	4
BGL-20 *d*_f_-4	BFRP	120	UHPC	Deep-ribbed	3
BGL-25 *d*_f_-4	BFRP	150	UHPC	Deep-ribbed	3
BGL-30 *d*_f_-4-1	BFRP	180	UHPC	Deep-ribbed	4
BGL-35 *d*_f_-4	BFRP	210	UHPC	Deep-ribbed	4
BSL-20 *d*_f_-3	BFRP	120	L-500	Deep-ribbed	3
BSL-25 *d*_f_-3	BFRP	150	L-500	Deep-ribbed	3
BSL-30 *d*_f_-3	BFRP	180	L-500	Deep-ribbed	3
BSL-35 *d*_f_-3	BFRP	210	L-500	Deep-ribbed	3
BSL-40 *d*_f_-3	BFRP	240	L-500	Deep-ribbed	3
BSY-20 *d*_f_-4	BFRP	120	L-500	Smooth	4
BSY-25 *d*_f_-4	BFRP	150	L-500	Smooth	4
BSY-30 *d*_f_-4	BFRP	180	L-500	Smooth	4
BSY-35 *d*_f_-4	BFRP	210	L-500	Smooth	4
CSL-40 *d*_f_-4	CFRP	240	L-500	Deep-ribbed	4
CGL-40 *d*_f_-4	CFRP	240	UHPC	Deep-ribbed	4
CGL-30 *d*_f_-4	CFRP	180	UHPC	Deep-ribbed	4
CSL-30 *d*_f_-4	CFRP	180	L-500	Deep-ribbed	4

**Table 2:** Static tensile results.

Specimen ID	Peak Load (kN)	Strength (MPa)	Failure Mode	Mean Strength (MPa)	Coefficient of Variation (%)
BRL-20 *d*_f_-3-1	22.11	778.49	Pullout	626.91	22.7
BRL-20 *d*_f_-3-2	14.23	495.40	Pullout		
BRL-20 *d*_f_-3-3	17.15	606.84	Pullout		
BRL-25 *d*_f_-3-1	25.54	903.75	Pullout	920.74	2.1
BRL-25 *d*_f_-3-2	26.50	937.72	Rupture		
BRL-25 *d*_f_-3-3	25.52	903.04	Rupture		
BRL-30 *d*_f_-3-1	27.91	983.79	Pullout	984.5	1.6
BRL-30 *d*_f_-3-2	27.84	985.21	Rupture		
BRL-30 *d*_f_-3-3	27.08	958.32	Rupture		
BRL-35 *d*_f_-3-1	28.25	999.65	Rupture	1014.27	2.0
BRL-35 *d*_f_-3-2	29.32	1037.51	Rupture		
BRL-35 *d*_f_-3-3	28.42	1005.66	Rupture		
BRL-40 *d*_f_-3-1	29.05	1027.95	Rupture	1082.93	5.5
BRL-40 *d*_f_-3-2	30.66	1084.92	Rupture		
BRL-40 *d*_f_-3-3	32.44	1147.91	Rupture		
BSL-20 *d*_f_-4-1	22.81	807.15	Rupture	\	\
BSL-25 *d*_f_-4-1	26.34	932.06	Rupture	\	\
BSL-30 *d*_f_-4-1	26.58	940.55	Rupture	\	\
BSL-35 *d*_f_-4-1	18.83	666.31	Rupture	\	\
BGL-20 *d*_f_-4-1	21.89	774.59	Rupture	\	\
BGL-25 *d*_f_-4-1	26.18	926.40	Rupture	\	\
BGL-30 *d*_f_-4-1	27.69	979.83	Rupture	993.63	7.1
BGL-30 *d*_f_-4-2	30.25	1070.42	Rupture		
BGL-30 *d*_f_-4-3	26.30	930.64	Rupture		
BGL-35 *d*_f_-4-1	21.46	759.38	Rupture	\	\
BSL-20 *d*_f_-3-1	25.15	889.95	Rupture	\	\
BSL-25 *d*_f_-3-1	28.97	1025.12	Rupture	\	\
BSL-30 *d*_f_-3-1	29.63	976.65	Rupture	1049.66	7.0
BSL-30 *d*_f_-3-2	29.63	1048.48	Rupture		
BSL-30 *d*_f_-3-3	31.76	1123.85	Rupture		
BSL-35 *d*_f_-3-1	20.99	742.75	Rupture	\	\
BSL-40 *d*_f_-3-1	24.7	874.03	Rupture	\	\
BSY-20 *d*_f_-4-1	26.98	954.71	Pullout	\	\
BSY-25 *d*_f_-4-1	23.06	815.99	Pullout	\	\
BSY-30 *d*_f_-4-1	13.05	461.78	Pullout	\	\
BSY-35 *d*_f_-4-1	20.83	737.08	Pullout	\	\
CSL-40 *d*_f_-4-1	\	\	Pullout	\	\
CSL-40 *d*_f_-4-2	10.2	\	Pullout		
CSL-40 *d*_f_-4-3	69.2	2448.69	Rupture		
CGL-40 *d*_f_-4-1	70.17	2483.01	Rupture	2375.21	4.3
CGL-40 *d*_f_-4-2	66.7	2360.23	Shear		
CGL-40 *d*_f_-4-3	64.5	2282.38	Shear		
CGL-30 *d*_f_-4-1	74.94	2651.80	Shear	2624.20	2.2
CGL-30 *d*_f_-4-2	75.22	2661.71	Shear		
CGL-30 *d*_f_-4-2	72.32	2559.09	Rupture		
CSL-30 *d*_f_-4-1	69.20	2448.69	Rupture	2477.71	1.8
CSL-30 *d*_f_-4-2	69.42	2456.48	Shear		
CSL-30 *d*_f_-4-3	71.44	2527.95	Rupture		

## Data Availability

Data are contained within the article.
